# Survival rates of head and neck cancers in Ghana: a retrospective study at the Komfo Anokye Teaching Hospital

**DOI:** 10.1186/s13104-020-05233-9

**Published:** 2020-08-26

**Authors:** Osei Owusu-Afriyie, W. K. B. A. Owiredu, Alexander Acheampong Oti, Emmanuel Acheampong, Kwabena Owusu-Danquah, Rita Larsen-Reindorf, Linda Ahenkorah Fondjo, Evans Asamoah Adu, Sampson Donkor, Peter Donkor

**Affiliations:** 1 Department of Molecular Medicine, School of Medical Science, Kwame Nkrumah University of Science and Technology, Kumasi, Ghana; 2 Department of Pathology, Komfo Anokye Teaching Hospital, Kumasi, Ghana; 3 Department of Medical Laboratory Technology, Faculty of Allied Health, Kwame Nkrumah University of Science and Technology, Kumasi, Ghana; 4 Directorate of Dental, Eye, Ear, Nose & Throat, Komfo Anokye Teaching Hospital, Kumasi, Ghana; 5 Department of Maxillofacial Surgery, Dental School, KNUST, Kumasi, Ghana; 6 School of Medical and Health Science, Edith Cowan University, Joondalup, Australia

**Keywords:** HNSCCs, Survival period, Conjunctiva, Oropharyngeal

## Abstract

**Objective:**

Data was collected to evaluate the survival rates of head and neck (conjunctiva, oropharyngeal and non-oropharyngeal) squamous cell carcinomas in Ghana.

**Data description:**

We provided data on a retrospective review of 8 years (January 2004 to December 2009) survival rate of head and neck squamous cell carcinomas (HNSCCs) at the Komfo Anokye Teaching Hospital in Ghana. The data consist of patient demographic data and clinicopathological findings which includes tumour site, tumour stage and histological grades of the patients. Clinical outcome measurement was death through to January 2013 on record and confirmed from the hospitals birth and death registry department. More than 85% of death cases were confirmed by gender, age, and folder identification numbers from the birth and death registry.

## Objective

Head and neck squamous cell carcinomas (HNSCCs) are heterogeneous tumours that develop in the oral cavity, oropharynx, hypopharynx, and larynx [1]. The incidence of HNSCCs vary broadly in Africa [2, 3] compared with that in western societies [4] largely because of wide variation in population size, economic status, ethnic origin, and belief in traditional medicine existing in Africa [5]. In Ghana, tumors of the pharynx and larynx represents 7.4% and 3.5% of all malignancies, and the second and seventh most common types of cancers, respectively seen at the National Hospital [6]. Also, data existing at the Komfo Anokye Teaching Hospital (KATH) indicate that tumors of the pharynx, larynx and oral cavity formed the largest group of HNSCCs, and most patients’ presents with late-stage disease [7, 8].

Despite recent advances in the diagnosis and treatment of head and neck cancer, there has been little evidence of improvement in survival rates over the last few decades [9]. Independent of the numerous reports on the epidemiology and molecular characteristics of HNSCCs in Ghana, there is a dearth of data on the survival rate of patients. The first attempt at population-based cancer registration was set up in 2012 however, it would take a few years to generate survival data. Identifying the need for survival analysis, we retrospectively compiled data on HNSCCs from the pathological perspective at KATH in Ghana over a period of 8 years.

## Data description

All patients referred to KATH or diagnosed by the multi-disciplinary team of doctors at the facility for HNSCCs are presented at a weekly meeting to the head and neck cancer clinic. As a routine management and monitoring plan, cases are discussed and a management plan decided upon. Data were obtained by retrospective review of all consecutive patient records seen at the multidisciplinary clinic from January 2004 to December 2009 and the survival probability data (Dataset 1) is included in Table 1 [10]. Demographic and basic health information was collected for all participants, along with pathology reports and tumor characteristics for HNSCC cases. Patient’s records were reviewed and staged according to the current World Health Organization’s International Classification of Disease coding system. Initial data obtained were evaluated for missing information and coded in Microsoft Excel sheet (Microsoft Office Professional Plus 2013). Overview of the Data file 1 [11] has been shown in Table 1.

**Table 1:** Overview of data files/data sets

Label	Name of data file/data set	File types (file extension)	Data repository and identifier (DOI or accession number)
Data file 1	General overview of data	MS Excel file (.xlsx)	Figshare (10.6084/m9.figshare.9878258.v2)
Data file 2	Cox regression analysis	MS Excel file (.xlsx)	Figshare (10.6084/m9.figshare.9878258.v2)
Figure 1	Survival analysis curves	tiff	Figshare (10.6084/m9.figshare.9878414.v1)
Data set 1	Excel sheet of data set on HNC cases from 2004-2009	MS Excel file (.xlsx)	Figshare (10.6084/m9.figshare.9878360.v2)

For clear and concise data that clearly answer our objectives, cases with lip cancer, substitute interview data, missing covariate information, distant metastasis, and site not otherwise specified were excluded. Therefore, we limited our data curation to 299 cases alive 1-year post-diagnosis. Outcome measurement was death through January 1, 2013 on record and confirmed from the hospitals birth and death registry department. More than 85% of death cases were confirmed by gender, age, and folder identification numbers from the birth and death registry. Survival function (Fig. 1) and cox regression analysis data (Data file 2) is also shown in Table 1 [11, 12]. The study was approved by the Committee on Human Research and Publication Ethics, Kwame Nkrumah University of Science and Technology as well as the Ethical committee board of KATH.

## Limitations

The data reflect specific patient population reporting to KATH, thus making it an institutional-based study and may not reflect the true picture of the situation in the entire Ghana population. In addition, the data does not constitute information on the treatment regimen, clinical symptoms, and socio-demographical characteristics, however age, gender and clinicopathological features were available for analysis.

## Data Availability

The data described in this data note is available and can be freely and openly accessed. Refer to Table 1 for the link to each data file; Data file 1 and 2 [11], Dataset 1 [10] Fig. 1 [12].

## Abbreviations

HNSCCs: Head and neck squamous cell carcinomas
KATH: Komfo Anokye Teaching Hospital
HR: Hazard ratio
